# Foreign Bodies in the Ear

**Published:** 1877-10-20

**Authors:** 


### FOREIGN BODIES IN THE EAR.

Dr. C. J. Blake, of Boston, in a paper read befor the American Medical Association, said:

"Cases in which a foreign body is lodged in the external auditory canal call perhaps more than any other accidents affecting this part of the organ of hearing for the exercise of patience and skill, the want of which often entails serious injury to the deeper seated and more delicate parts of the organ of hearing. Voltolini pithily says that the point of danger in the external auditory canal will do much less harm than injudicious attempts at its removal. The latter text-books decry any attempts made for the removal of foreign bodies from the ear that are not made with a full knowedge of the structure and relations of the parts of the ear in question, of the character and location of the foreign body, and of the various methods which may be employed for its removal. The paper here contains a brief review of the topography of the external ear. No attempt at removal should be made without proper and sufficient illumination of the parts. The body of the membrana tympani is formed by two layers of fibrous tissue, the fibres of which are so arranged as to give it great strength and elasticity, but not sufficient strength to justify its use as a point of resistance in the attempt at instrumental removal of a foreign body which may be lodged against it.

" Cases have come under the author's observation in which, for the want of an observance of these precautions, the membrana tympani had been ruptured, and the malleus torn away. In the majority of cases, even including those in which the foreign body may be firmly impacted, the removal may be effected with little or no pain to the patient, the force used being expended upon the entire surface of the foreign body without using the walls of the canal or the membrana tympani as points of resistance, or indirectly upon the inner surface of the foreign body. Pain, as a rule, implies injury, which might be avoided by patient and delicate manipulation. Slight injury may entail serious consequences to the health of the patient. Since bodies which find their way into the ear vary, the means employed for their removal must therefore vary, and the choice will depend upon the judgment of the surgeon and the means at his command.

"The cases which present the g eatest difficulties are those of hard bodies, such as stones, beads, buttons, and the like bodies liable to expand by the absorption of moisture, such as beans and peas and impacted masses of epidermis, which resent the action of water and offer no hold for the forceps or hook

" Out of 2,374 cases examined during the past year, in the aural clinic of the Massachusetts Charitable Eye and Ear Infirmary, there were thirty cases of foreign bodies; of this number fourteen were cases in which a bean, pea, kernel of corn, or similar substance, had been pushed into the ear. In six cases insects were removed; and in one case the living larvae of the common blow-fly. In one of the common cases, a bean had remained ten weeks without causing irritation; in another, a small bean had been pushed into the middle-ear in consequence of a previous attempt at its extraction. In the majority of the cases the foreign body was removed by means of syringing with warm water, which possesses the advantage of permitting the application of the maximum of force with the minimum of danger, the force moreover being applied without the foreign body. The object to be accomplished in the use of the syringe is to establish a body of water between the foreign body and the membrani tympani, connected by a slender column of water between the foreign body and the wall of the canal with the column of water in the syringe, furnishing in this manner the elements of a hydraulic press.

" Among the most simple procedures, that of Dr. Lowenberg, of Paris, may sometimes prove useful. This consists in the application of a camel's-hair pencil, dipped in joiner's glue, to the foreign body, which should first be carefully dried by wiping with absorbing cotton. The glue is allowed to set, and the removal then effected."—*Chicago Med. Journal and Examiner.*

